# Antistress Effects of the Ethanolic Extract from *Cymbopogon schoenanthus* Growing Wild in Tunisia

**DOI:** 10.1155/2013/737401

**Published:** 2013-10-08

**Authors:** Mahmoud Ben Othman, Junkyu Han, Abdelfatteh El Omri, Riadh Ksouri, Mohamed Neffati, Hiroko Isoda

**Affiliations:** ^1^Graduate School of Life and Environmental Sciences, University of Tsukuba, 1-1-1 Tennodai, Tsukuba City, Ibaraki 305-8572, Japan; ^2^Faculty of Life and Environmental Sciences, University of Tsukuba, 1-1-1 Tennodai, Tsukuba City, Ibaraki 305-8572, Japan; ^3^Alliance for Research on North Africa (ARENA), University of Tsukuba, 1-1-1 Tennodai, Tsukuba City, Ibaraki 305-8572, Japan; ^4^Laboratoire d'Adaptation des Plantes aux Stress Abiotiques, Centre de Biotechnologie à la Technopole de Borj-Cédria (CBBC), BP 901, 2050 Hammam-lif, Tunisia; ^5^Arid Lands Institute, Range Ecology Laboratory, 4119 Medenine, Tunisia

## Abstract

This study aimed to investigate the antistress properties of the ethanol extract of *Cymbopogon schoenanthus* (CSEE), growing wild in the southern part of Tunisia. The effect of extracts on H_2_O_2_-induced cytotoxicity and stress in human neuroblastoma SH-SY5Y cells. Its effect on stress-induced in ICR mice was exposed to force swim and tail suspension, in concordance with heat shock protein expression (HSP27 and HSP90), corticosterone, and catecholamine neurotransmitters level. Our results demonstrated that pretreatment of SH-SY5Y cells with CSEE at 1/2000, 1/1000, and 1/500 v/v dilutions significantly inversed H_2_O_2_-induced neurotoxicity. Moreover, CSEE treatments significantly reversed heat shock protein expression in heat-stressed HSP47-transformed cells (42°C, for 90 min) and mRNA expression of HSP27 and HSP90 in H_2_O_2_-treated SH-SY5Y. Daily oral administration of 100 mg/kg and 200 mg/kg CSEE was conducted to ICR mice for 2 weeks. It was resulted in a significant decrease of immobility time in forced swimming and tail suspension tests. The effect of CSEE on animal behavior was concordant with a significant regulation of blood serum corticosterone and cerebral cortex levels of catecholamine (dopamine, adrenaline, and noradrenaline). Therefore, this study was attempted to demonstrate the preventive potential of CSEE against stress disorders at *in vitro* and *in vivo* levels.

## 1. Introduction 

Stress is known to induce alterations in various physiological responses even leading to pathological states [1]. It was demonstrated that different stress paradigms [2, 3] significantly affected learning and memory function and intensified fear memory in mice. The effects are supposed to be an outcome of a complex interaction of stress and altered activity of different mechanisms such as decrease in central neurotransmitters, neurohormonal factors [4], and neurotrophic factors [5] and increase in free radical generation and oxidative damage in the central nerve system [6].

In view of the potential use of natural products and botanicals as stress adaptogens, antioxidant rich phytochemical extracts are gaining a lot of interest. In this respect, several plants are traditionally and clinically used for the management of neurological disorders. From our previous research and others, several plants and herb preparations were demonstrated to protect against neuronal damage [7], Parkinson and Alzheimer' disease [8, 9], to improve neuronal differentiation [10], and to act against depression [11, 12], epilepsy [13], and anxiety [14]. 

Recent surveys reported that psychiatric conditions especially stress and depression were among the most common mood pathologies treated with complementary and alternative therapies [15, 16]. This correlates with a worldwide increasing trend to integrate traditional medicine with primary health care, because of its “green image”, its cultural significance, and its accessibility to all societal categories [17]. 

Herbal medicines are an important part of the culture and traditions in the African continent where around 122 drugs originating from 94 species have been discovered through ethnobotanical leads [18]. North Africa and Sahara are known by their richness in medicinal plants which are gathered and used as greens, spices, and condiments [19]. About 70% of the wild plants in North Africa are known to be of potential value in fields such as medicine and biotechnology.

Tunisia has a large plant biodiversity. Actually, over 350 species are considered herbal, medicinal, and aromatic plants (HMAP). These resources are used, mainly by rural communities for traditional phytotherapy, and practiced for several generations and civilizations [20]. Preservation of these species and knowledge of their uses require specific intervention, and these resources should not be lost due to environmental degradation, agricultural expansion, and urbanization. In this respect, this study was conducted to elucidate the traditional usage of *Cymbopogon schoenanthus* L. spring (CS). 

CS is an aromatic culinary herb grown in the southern area of Tunisia, and it is locally known as “El bekherai”. It is used for several preparations of meat and salad or served with tea because of its pleasant aroma appreciated by north African inhabitants [21]. Besides its use in culinary preparations, CS is also used in folk medicine. Its decoction and infusion are taken as diuretic to reduce intestine spasm and to act against food poisoning, antirheumatism, antianorexia, and digestive [22]. Our recent research demonstrated that CS extracts and essential oil have antioxidant and acetylcholinesterase inhibitory properties [23, 24].

Using *in vitro* bioassay and *in vivo *models, the antistress effect of ethanol extract of *Cymbopogon schoenanthus* (CSEE) was evaluated with a focus on the heat shock protein role in protecting neuronal cells against H_2_O_2_-induced cytotoxicity in SH-SY5Y cells, corticosterone, and catecholamine regulated levels in stressed-mice. Human neuroblastoma SH-SY5Y cells and HSP47-transformed cells were used as *in vitro* model; tail-suspended and forced-swim ICR mice were used as *in vivo *model.

## 2. Materials and Methods

### 2.1. Plant Extracts Preparation

*C. schoenanthus* was collected from the south of Tunisia in February 2011. The leaves were allowed to dry on the shadow at 25°C, and then they were ground into fine powder. 100 g of the powdered leaves was soaked in 1000 mL of ethanol (70%), for 2 weeks. The liquid fraction was centrifuged, filtered at 0.22 *μ*m, and kept at −80°C.

### 2.2. HPLC Analysis of CSEE

Diluted samples were injected to RP-HPLC. The separation of phenolic compounds was performed with an Agilent 1100 series HPLC system equipped with online degasser (G 1322A), quaternary pump (G 1311A), a thermostatic autosampler (G 1313A), column heater (G1316A), and diode array detector (G 1315A). Instrument control and data analysis were carried out using Agilent HPLC Chemstation 10.1 Edition of Windows 2000. The separation was carried out on a reverse phase ODS C18 (250 mm × 4.6 mm ID, 5 *μ*m particle size Hypersil) column used as stationary phase at ambient temperature. The mobile phase consisted of acetonitrile (solvent A) and water sulphuric acid (0.2%) (Solvent B). The flow rate was kept at 0.5 mL·min^−1^. The gradient program was as follows: 15% of A/85% of B at 0–12 min, 40% of A/60% of B at 12–14 min, 60% of A/40% of B at 14–18 min, 80% of A/20% of B at 18–20 min, 90% of A/10% of B at 20–24 min, and 100% of A at 24–28 min. The injection volume was 20 *μ*L, and peaks were monitored at 280 nm. Peak identification was obtained by comparing the retention time and the UV spectra of sample phenolic chromatogram with those of pure standards from Sigma (St. Louis, MO, USA). Analyses were performed in triplicates.

### 2.3. Cell Culture

Human neuroblastoma SH-SY5Y cells were cultured in 1 : 1 mixture of Dulbecco's minimum essential medium (DMEM; Sigma, USA) and Ham's F-12 nutrient mixture (Sigma, USA) supplemented with 15% fetal bovine serum (FBS; Sigma, USA), 1% nonessential MEM amino acid, and 1% penicillin (5000 *μ*g/mL)-streptomycin (5000 IU/mL) solution (ICN Biomedical, Inc.). The cells were cultured in 100 mm dishes and passaged by trypsinization using 0.25% Trypsin-EDTA (Sigma) at 80% confluence twice a week. Medium was changed every other day, and cells were incubated at 37°C (5% CO_2_).

Chinese hamster ovary (CHO) cells, stably transfected with (+) or without (−) HSP47 promoter, were cultured in F12 medium supplemented with 10% FBS, 0.2% Kanamycin solution, and 0.1% G418 (Gibco BRL 13075-015). The cells were cultured in 75 cm^2^ T-flasks and passaged by trypsinization at 80% confluence. Medium was changed every two days, and cells were incubated at 37°C (5% CO_2_).

### 2.4. Neuroprotective Properties of CSEE against H_2_O_2_-Induced Cytotoxicity in SH-SY5Y Cells

SH-SY5Y cells were seeded at 1 × 10^4^ cell/well in 96 well microplates and allowed to attach for 24 h incubation. Then cells were treated with CSEE (1/100, 1/1000, and 1/10000) for 72 h to investigate the noncytotoxic concentrations or in combination with H_2_O_2_. In H_2_O_2_ treatment panel, SH-SY5Y cells were pretreated with CSEE at 1/2000, 1/1000, and 1/500 for 24 h, followed by treatment with 150 *μ*M H_2_O_2_ for 24 h. Cell viability was performed using 3-(4, 5-Dimethylthiazol-2-yl)-2, 5-diphenyltetrazolium bromide (MTT) as explained in our previous research [10]. Briefly, 10 *μ*L of MTT solution (5 mg/mL in PBS (−)) was added to each well of 96-well plates and incubated for 6 h at 37°C in a 95% humidified air—5% CO_2_ incubator. Then the formazan was formed and later dissolved in 10% sodium dodecyl sulfate (SDS). The absorbance was determined at 570 nm using blanks (wells which contain a mixture of extract and medium without cells) as a reference. Cell viability was reported as a percentage of control cells (cells treated with medium only).

### 2.5. HSP47 Assay

HSP47-transformed cells were plated at 1 × 10^4^ cells/well in 100 *μ*L of culture medium and allowed to attach and grow for 48 h at 37°C (5% CO_2_). Then, the cells were subjected to a heat shock for 90 min at 42°C, and recovered at 37°C during 2 h. After the recovery time, cells were treated with CSEE (1/1000, 1/500, and 1/100 v/v dilutions) and incubated for 3 h at 37°C, in 5% CO_2_ incubator. Then the cells were washed twice with PBS (−), and HSP47 assay was performed as described in our previous study [25]. Moreover, the effect of CSEE on HSP47-transformed cell viability was investigated using MTT assay as previously described.

### 2.6. Effect of CSEE on HSP27 and HSP90 mRNA Expression in H_2_O_2_-Treated SH-SY5Y Cells

SH-SY5Y cells were plated at 2 × 10^5^ cells/mL in 100 mm dish and allowed to attach for 24 h, then treated with various concentrations of CSEE at 1/500, 1/1000, and 1/2000 for 24 h, and then cotreated with 150 *μ*M H_2_O_2_ for 24 h. Cells were washed with cold PBS, and total RNA was extracted using Isogen Kit (Wako, Japan). Total RNA was quantified by Thermo scientific Nano drop 2000 (USA). Reverse transcription reactions were performed using the Superscript III reverse transcriptase kit (Invitrogen, Carlsbad, CA, USA) [11].

All Primer sets: HSP27 (Hs03044127_g1), HSP90 alpha (Hs00743767_sH), and GAPDH and TaqMan probes for experimental genes were analyzed using Applied Biosystems. HSP27 and HSP90 mRNA expression was quantified using TaqMan real-time quantitative PCR (AB 7500 fast real-time system, Applied Biosystems, U.S.A.). Amplifications were performed in 20 *μ*L final volume as explained in our previous study [11]. Gene expression was normalized to GAPDH and reported as fold of control.

### 2.7. Animal Treatment

Sixty-four ICR mice weighing between 26–30 g at 5 weeks of age (Charles River, Japan) were used for *in vivo *experiments. The animals were individually housed and allowed to acclimatize with free access to food and water for extra one week under a 12/12 light dark cycle, with controlled temperature (23°C) and humidity (40–60%). After acclimatization, animals were assigned to 2 groups of 32 animals each that were used respectively for TST and FST. Each group was randomized into 4 subgroups of 8 animals each: (1) vehicle control group (administered with distilled water), (2) imipramine group (administered with 15 mg/kg imipramine), (3) CSSE 100 group (administered with 100 mg/kg CSSE), and (4) CSSE 200 group (administered with 200 mg/kg CSSE).

Imipramine and *C. schoenanthus* dried ethanol extract were freshly dissolved in distilled water and were orally administered (p.o) to mice in a volume of 10 mL/kg body weight daily for 13 consecutive days. One hour prior to extract and drug administration, all mice were deprived from food but not water; in other time periods, all animals had free access to food and water [26].

### 2.8. Tail Suspension Test

The tail suspension test (TST) was based on the method of Steru et al. [27] with minor modifications. Briefly, all mice were individually suspended by the tail with clamp (1 cm distant from the top) for 6 min in a box (35 × 70 × 50 cm) with the head 5 cm to the bottom. Testing was carried out in a darkened room with minimal background noise, 1 h after plant extract and 30 min after imipramine administration. The duration of immobility time was considered during the final 3 min interval.

### 2.9. Forced Swimming Test

The forced swimming test (FST) was conducted as previously described by Porsolt et al. [28]. Briefly, each mouse was placed in a 25 cm glass cylinder (14 cm diameter) filled with water up to 15 cm, maintained at 24 ± 2°C, and forced to swim for 6 min (before the swimming session). Then mice were removed and dried. They were again forced to swim in a similar environment for a period of 6 min and 24 h later (test session). Immobility duration was recorded using a camera during the last 3 min of the 6 min tests. Extracts were administered 1 h before the forced swimming test, or imipramine was administered 30 min before the test. In the subchronic treatment study, the same dosages of extracts or imipramine, as those in the acute treatment study, were administered once a day for 2 weeks, and the final treatment was conducted 1 h (extracts) or 30 min (imipramine) before behavioral tests.

### 2.10. Determination of Corticosterone in CSEE-Treated ICR Mice Blood Serum

Serum corticosterone (CORT) levels in the control and CSEE-treated mice were determined using an enzyme immunoassay kit (AssayMax Corticosterone ELISA Kit, AssayPro LLC) according to manufacturer's recommendations. Blood serum was collected by centrifugation at 3000 xg for 10 min, then it was diluted at 1/200 with EIA diluent and immediately stored at −20°C until use. 25 *μ*L of different serum samples or standard solution was mixed with 25 *μ*L of biotinylated corticosterone in each well and allowed to stand for 2 h at room temperature. Then wells were washed and incubated with 50 *μ*L of streptavidin-peroxidase conjugate for 30 min; afterwards, wells were washed again and incubated with 50 *μ*L of chromogen substrate solution for 30 min until the optimal blue color density develops. Then, 50 *μ*L of stop solution was added to each well, and finally, the absorbance in each well was recorded at 450 nm. The level of CORT was also calculated using standard curve and reported in ng/mL.

### 2.11. Determination of Dopamine (DOP), Adrenaline (ADR), and Noradrenaline (NAD) in CSSE-Treated ICR Mice Brain Tissue

Cerebral cortex catecholamine (Dopamine (DOP), adrenaline (ADR), and noradrenaline (NAD)) levels were determined using 3-CAT Research ELISA (Labor Diagnostika Nord GmbH & Co. KG) according to manufacturer's instructions. Briefly, 100 mg of brain tissue was homogenised. Also 60 *μ*L of brain sample or standards were mixed with 25 *μ*L of TE buffer into all wells, and the plate was shaken for 1 h at room temperature. After washing twice, 150 *μ*L of acylation buffer and 25 *μ*L of acylation reagent were added to each well. Then wells were shaken, washed, and incubated with 200 *μ*L of hydrochloric acid for 10 min at room temperature. 190 *μ*L from each well were taken and incubated for 2 h at 37°C with 50 *μ*L of enzyme solution. Afterwards, 75 *μ*L of samples from enzyme plate were mixed with 50 *μ*L of the respective antiserum into all wells and incubated for 18 h at 4°C. Plates were incubated for 30 min at room temperature after washing and adding 100 *μ*L of enzyme conjugate in each well. Then, wells incubated with 100 *μ*L of substrate for 30 min at room temperature and 50 *μ*L of stop solution was added. Finally, the absorbance in each well was recorded at 450 nm and the level of catecholamine was calculated using standard curve and reported in ng/mL.

### 2.12. Statistical Analysis

All data were expressed as mean ± S.E.M, and significances were calculated using Student's *t*-test. Statistical significance was set at *P* < 0.05.

## 3. Results

### 3.1. HPLC Analysis

Seven phenolic compounds were successfully identified in CSEE based on the retention time and spectral characteristics of their peaks against those of the standards using RP-HPLC coupled with UV-Vis multiwave length detector. The chromatogram of plant extract was compared to authentic standards of phenolic acid and flavonoid profiles, which allowed us to identify 5 phenolic compounds at 330 nm: quercetin-3-rhamnoside, trans-cinnamic acid, resorcinol, caffeic acid, and 2.5-dihydroxybenzoic acid and 2 others phenolic compounds at 280 nm: ferulic acid and gallic acid (Table 1).

### 3.2. CSEE Protects SH-SY5Y Cells against H_2_O_2_-Induced Toxicity

**Figure 1:**
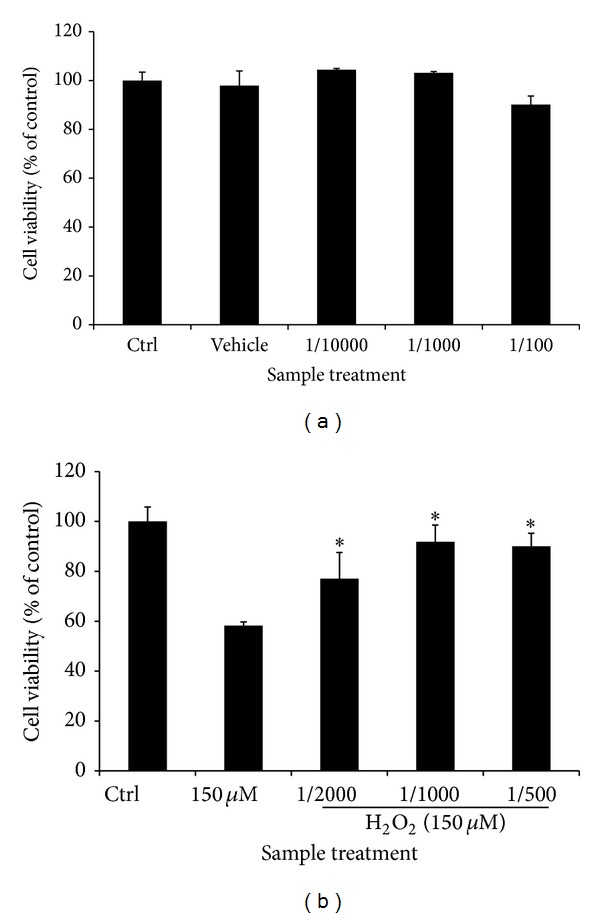
*Neuroprotective properties of CSEE against H*_2_ *O*_2_ *-induced cytotoxicity*. SH-SY5Y cells were seeded at 1 × 10^4^ cell/well in 96-well microplate and treated with (a) CSEE (1/10000, 1/1000, and 1/100 v/v dilutions) for 72 h, (b) SH-SY5Y cells were pretreated with CSEE (1/10000, 1/1000 and 1/500 v/v dilutions) for 24 h and then treated with 150 *μ*M H_2_O_2_ for 24 h. Cell viability was determined using MTT assay as explained in Materials and Methods. Each bar represents the mean of 3 independent trials ± SD. *P < 0.05, **P < 0.01 versus control cells, (Student's *t*-test).

To determine the noncytotoxic concentrations of CSEE, SH-SY5Y cells were treated with CSEE at 1/10000, 1/1000, and 1/100 v/v dilution for 72 h. CSEE did not affect SH-SY5Y cell viability. However at 1/100 dilution cell viability slightly decreased, but this decrease was not significant (Figure 1(a)).

In preliminary experiments, 150 *μ*M H_2_O_2_ was found to be the challenging concentration to reduce SH-SY5Y cells by 40% after 24 h exposure (data not shown). Pretreatment of SH-SY5Y cells with CSEE (1/2000, 1/1000, and 1/500 v/v dilutions) significantly and dose dependently improved cell viability in H_2_O_2_-treated cells to reach 77 ± 8.53%, 91 ± 6.66%, and 90 ± 5.19% of control, respectively.

### 3.3. CSEE Reduce Heat Stress Effect in HSP47 Transformed Cells

**Figure 2:**
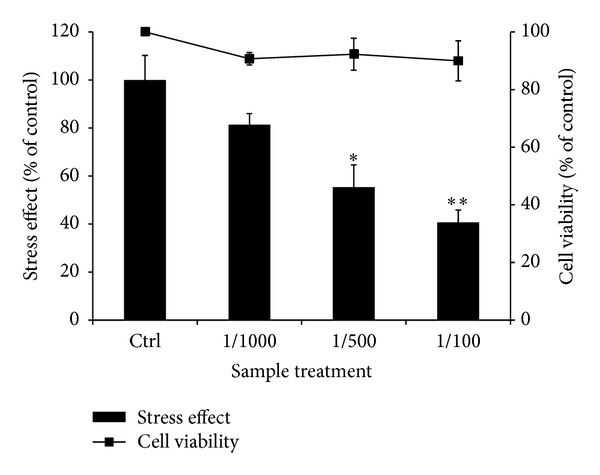
*Effect of CSEE on HSP47 expression and cell viability in Chinese hamster ovary transformed cells*. Cells were seeded at 1 × 10^4^ cell/well in 96-well microplate and treated with CSEE at 1/1000, 1/500, 1/100 v/v dilution. Cell viability and HSP47 expression were performed as explained in Materials and Methods. Each bar represents the mean of 3 independent trials ± SD. *P < 0.05, **P < 0.01 versus control cells, (Student's *t*-test).

To investigate the antistress effect of CSEE, HSP47-transformed cells were used as model. Cells were heat-shocked for 90 min at 42°C, 5%CO_2_ and recovered for 2 h at 37°C, 5%CO_2_. Then cells were exposed to CSEE at 1/1000, 1/500, and 1/100 v/v dilutions for 3 h. CSEE significantly and dose dependently reduced HSP47 expression by 81.4 ± 4.64%, 55.36 ± 9.28%, and 40.7 ± 5.11% of control, respectively without cytotoxic effect (Figure 2).

### 3.4. CSEE Pretreatment Reduces the H_2_O_2_-Stimulated mRNA Expression of HSP27 and HSP90 in SH-SY5Y Cells

**Figure 3:**
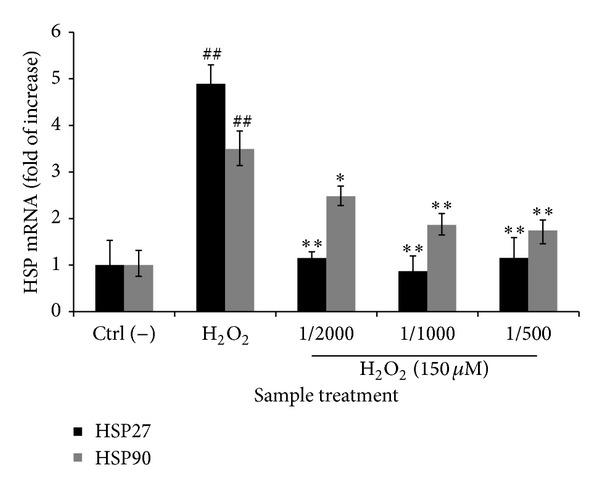
*Effect of CSEE on mRNA expression of HSP27 and HSP90 in H*_2_ *O*_2_ *-treated SH-SY5Y cells*. SH-SY5Y cells were seeded at 2 × 10^5^ cell/mL in 100 mm dish and was treated with CSEE (1/500, 1/1000 and 1/2000) for 24 h, and then they were treated with 150 *μ*M H_2_O_2_ for 24 h. The mRNA expression of genes was normalized to GPDH mRNA expression and expressed as ratio of control. Each bar represents the mean of duplicate ± SD. *P < 0.05, **P < 0.01 versus positive control group, and ^#^ *P* < 0.05, ^##^ *P* < 0.01 versus control group (Student's *t*-test).

To assess the mechanism by which CSEE reversed H_2_O_2_-induced toxicity, we investigated the effects of CSEE pretreatments on heat shock proteins HSP27 and HSP90 mRNA expression in H_2_O_2_-treated SH-SY5Y cells. H_2_O_2_ treatment increased HSP27 and HSP90 by 5 ± 0.40 and 3.5 ± 0.38 fold of control, respectively. When pretreated with CSEE (1/2000, 1/1000, and 1/500) for 24 h, HSP27 mRNA expression significantly decreased to reach the control level. HSP90 mRNA significantly decreased only in 1/1000 and 1/500 dilution treatments to reach 2 ± 0.21 fold of control (Figure 3).

### 3.5. CSEE Oral Administration Reduced Immobility Time in the TST and in the FST

CSEE at 100 and 200 mg/kg (p.o) caused neither the death of any animal nor the change in mice coat color. Moreover, CSEE doses (p.o) did not reduce body weight in TST or FST significantly (data not shown). 

The antistress effect of orally administered CSEE in ICR mice was investigated for 13 consecutive days by recording immobility time in TST and FST.

**Figure 4:**
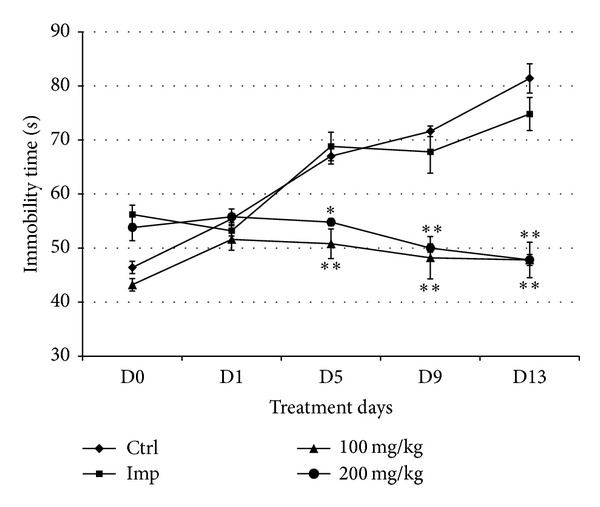
*Effect of administration of CSEE on the immobility time in the TST. *ICR mice were orally administrated with distilled water in control group, 15 mg/kg imipramine, and 100 and 200 mg/kg CSEE. The immobility time in the TST was calculated as reported in Materials and Methods. Data are represented as the average of 5 observations ± SEM. *P < 0.05 versus Control group (Student's *t*-test).

The baseline of immobility time (day 0, prior to CSEE (p.o)), shows 2 clusters (Figure 4). However, on day 1, the immobility time did not show any significant difference among the four animal groups. The TST was assessed every four days, and starting from day 5 the immobility time was increased in control and imipramine groups to reach 81 ± 2.7 sec and 75 ± 3.0 sec on day 13. In CSEE-treated groups, 100 mg/kg and 200 mg/kg doses significantly maintained the immobility time in ICR mice shorter than that in vehicle and imipramine groups during all the period of the experiment. In fact, both 100 and 200 mg/kg CSEE resulted in 48 ± 2.5 sec on day 13 with a similar trend. The effects of CSEE appeared to be more potent than those of imipramine during the last days of treatment.

**Figure 5:**
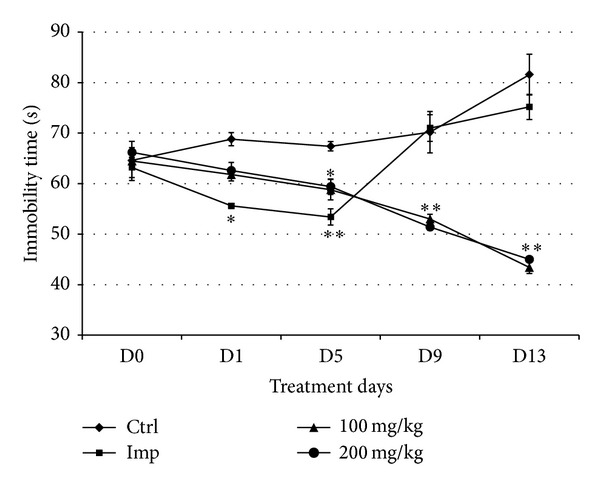
*Effect of administration of CSEE on the immobility time in the FST*. ICR mice were orally administered with distilled water in control group, 15 mg/kg imipramine in positive control group, and 100 and 200 mg/kg of CSEE. The immobility time in the FST was calculated as reported in Materials and Methods. Data are represented as the average of 5 observations ± SEM. *P < 0.05 versus control group (Student's *t*-test).

Similarly to TST, FST was assessed every four days during 2 weeks. The immobility time was increased in the control group to reach 82 ± 4 sec in the last day of treatment. In imipramine group, the immobility time was decreased to reach 53 ± 1.6 sec on day 5, then it was increased to reach 75 ± 2.4 sec on the day 13 (Figure 5). However, CSEE at 100 and 200 mg/kg significantly reduced the immobility time to reach 45 ± 0.8 sec on D13. 

### 3.6. Effects of CSEE on Serum Corticosterone Levels

**Figure 6:**
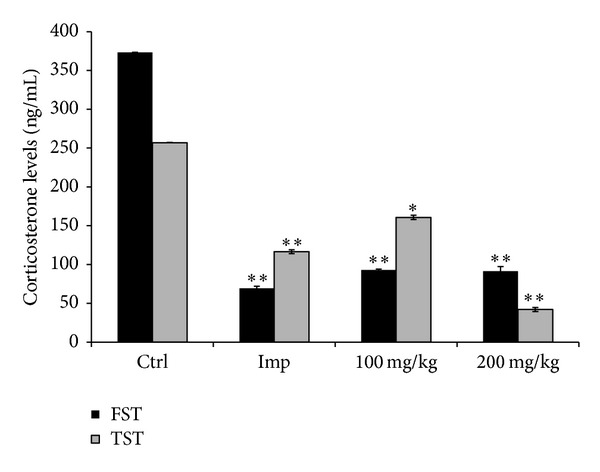
*Effect of CSEE on serum level of corticosterone in ICR mice subjected to FST and TST*. Mice fed with 100 and 200 mg/kg of CSEE for 14 days were sacrificed by spinal cord dislocation one day after the last behavior test; then the blood sample was collected, and the serum was rapidly separated. Plasma corticosterone was measured using Assay Max Corticosterone ELISA kit as explained in Materials and Methods. Each value represents the mean of ±SD (*n* = 4). *P < 0.05 versus control group (Student's *t*-test).

The swim stress procedure evoked a significant increase in serum CORT levels 373 ± 0.3 ng/mL in the control group (Figure 6). CSEE, at 100 mg/kg and 200 mg/kg, and imipramine (15 mg/kg) significantly reduced the serum CORT levels in FST-induced stress in mice to 92 ± 1.3, 91 ± 3.5, and 69 ± 2.7 ng/mL, respectively.

Similarly with FST, a significant increase in serum CORT concentrations was observed in control mice exposed to TST. Further, 100 and 200 mg/kg of CSEE and imipramine (15 mg/kg) significantly reduced the serum CORT levels in mice to 160 ± 2.7, 42 ± 2.5, and 116 ± 2.5 ng/mL, respectively (Figure 6).

### 3.7. Effect of CSEE on Monoamine Neurotransmitters Levels in Brain

DOP, ADR, and NAD were significantly decreased to 5.1 ± 0.07, 0.3 ± 0.05, and 25.2 ± 0.04 ng/mL, respectively, in stressed mice when exposed to tail suspension test (Table 2). CSEE at 100 mg/kg and 200 mg/kg and imipramine (15 mg/kg) significantly increased the DOP, ADR, and NAD levels (Table 2). A significant increase of monoamine neurotransmitter levels was observed in treated groups' brain in FST. However, in control group, DOP, ADR, and NAD were significantly decreased to 6.1 ± 0.02, 0.2 ± 0.07, and 45.6 ± 0.03 ng/mL respectively.

## 4. Discussion

The present study investigated the potential preventive effect of CSEE against stress in H_2_O_2_-treated SH-SY5Y cells, in heat-stressed HSP47-transformed cells, and in two mouse models of despair tasks: the TST and the FST. Moreover, neurobiochemical and neuropharmacological paradigms were used as tools to contribute to the understanding of the involvement of heat shock proteins (HSP27 and HSP90), catecholamines neurotransmitters (dopamine, adrenaline, and noradrenaline), and corticosterone in the antistress-like activity of CSEE.

It is well known that chronic exposure to stress is causative factor of free radical generation and reactive oxygen species elevation in the body [6]. Oxidative stress is well demonstrated to be a leading factor in neuronal cell death and damage [29]. Thus, antioxidants intake might be an effective strategy to protect the human body against stress-mediated pathologies. In this respect, phytochemicals, botanicals, and plant extracts are gaining a lot of interest as complementary supplements to fight against oxidative stress. In the current study, CSEE, a polyphenol-rich plant extract, treatment showed a significant protective effect against H_2_O_2_-induced toxicity in SH-SY5Y cells and against heat stress-induced in HSP47-transformed cells. Moreover, CSEE significantly reversed HSP27 and HSP90 mRNA expressions in H_2_O_2_-treated SH-SY5Y cells.

A probable underlying mechanism of this protection may be associated with the presence of flavonoids (Resorcinol and Quercetin-3-rhamnoside) and phenolic acids (Trans-cinnamic acid, Ferulic acid and Gallic acid). In our study, we demonstrated that CSEE protection against H_2_O_2_-induced stress was regulated by heat shock proteins (HSP). In fact, HSPs provide a fundamental mechanism to defend neuronal cells against the effect of diverse stressors like temperature, oxidation, inflammation, xenobiotics, irradiation, and pollutants [25]. HSPs are becoming a therapeutic target in neurodegenerative diseases and aging because the pathogenesis mechanism of these diseases is thought to be related to an abnormal increase of unfolded protein response [30]. Beside its role against protein aggregation, HSP27 is reported to have the capacity to sequester cytochrome C when released from the mitochondria into the cytosol and to have important antioxidant properties [31] by decreasing the abundance of reactive oxygen species (ROS) [32] and maintaining glutathione in its reduced form [33]. Moreover, HSP27 is involved in the cell survival mechanism since its depletion was demonstrated by Rocchi et al. [34] to result in cell apoptosis through caspase3 activation. On the other hand, it was reported that HSP90 acts on misfolded proteins induced by conditions such as heat and the presence of ROS. In addition, in the absence of HSP90, many clients are turned over when misfolded or misassembled [35]. Same authors suggested that “stress” moves cells away from homeostasis and leads to an increase of the load for HSP90 and its co-chaperones.

Since the first emergence of “stress”, researchers tried to find and develop several animal models to solve this problem. The tail suspension and forced swimming tests are the most common predictive tests for screening the beneficial properties of medicinal plants [27, 28]. 

In traditional medicine, *C. schoenanthus* was used for several treatments. However, its antistress effect is still not yet established *in vivo*. Within the same genus of *Cymbopogon, C. citratus* essential oil was reported to have an anxiolytic activity [36, 37]. Moreover, Quintans-Júnior et al. [38] demonstrated the anticonvulsant effect of the essential oil from *C. winterianus*. The present study was the first to demonstrate that oral administration of *C. schoenanthus* ethanol extract (100 and 200 mg/kg, p.o.) significantly reduced immobility time in the TST and FST in mice compared to vehicle group and the classical tricyclic antidepressant, imipramine (15 mg/kg, p.o.) treated group. The immobility time in TST and FST, referred to behavioral despair in animals, is believed to reproduce a condition similar to human depression [39]. Thus, a reduction in the total duration of immobility indicates an antistress effect [27, 40]. Moreover, the effect of CSEE on animal behavior was concordant with its effect on blood corticosterone and cerebral cortex monoamine levels. In fact, CSEE oral administration at 200 mg/kg significantly decreased corticosterone levels to 42 ± 2.5 ng/mL and improved DOP, ADR, and NAD to 6.6±, 0.6±, and 53.7± ng/mL, respectively. In this respect, from our and others research, there is a great interest in phytochemicals and dietary molecules that may interact with the hypothalamic-pituitary-adrenal (HPA) axis and the mono-amenergic, catecholaminergic, and cholinergic systems [11, 41]. The precise mechanisms by which CSEE produced its antistress effects were not fully understood. However, according to our results, this effect could be regulated by its interaction with the monoaminergic system and HPA axis. In this context, it is already suggested that the first step of antistress effect mechanism should be considered as an increase in the monoamine levels at the synapse [42] and a decrease in corticosterone serum levels [43].

Taken together, our results demonstrated that CSEE was effective in producing significant antistress effects at *in vitro* and *in vivo *levels. The molecular mechanism by which CSEE exhorted its beneficial effects seems to be partially modulated by chaperone activation, monoaminergic system, and HPA axis regulation. Furthermore, the *in vivo *results of CSEE oral administration showed a comparable effect to the established commercial antidepressant drug (imipramine). The decrease of immobility time was dose dependent in two models. These results indicated that CSEE had a dose-dependent antistress effect that was comparable to established commercial antidepressant drugs.

The HPLC analysis of CSEE identified several compounds with known neuroprotective activities like caffeic acid, ferulic acid, and quercetin. However, future studies should be addressed in exploring their mixture and the molecular mechanism that may regulate their activities.

## Figures and Tables

**Table 1:** Major compounds of *C. schoenanthus* identified by HPLC.

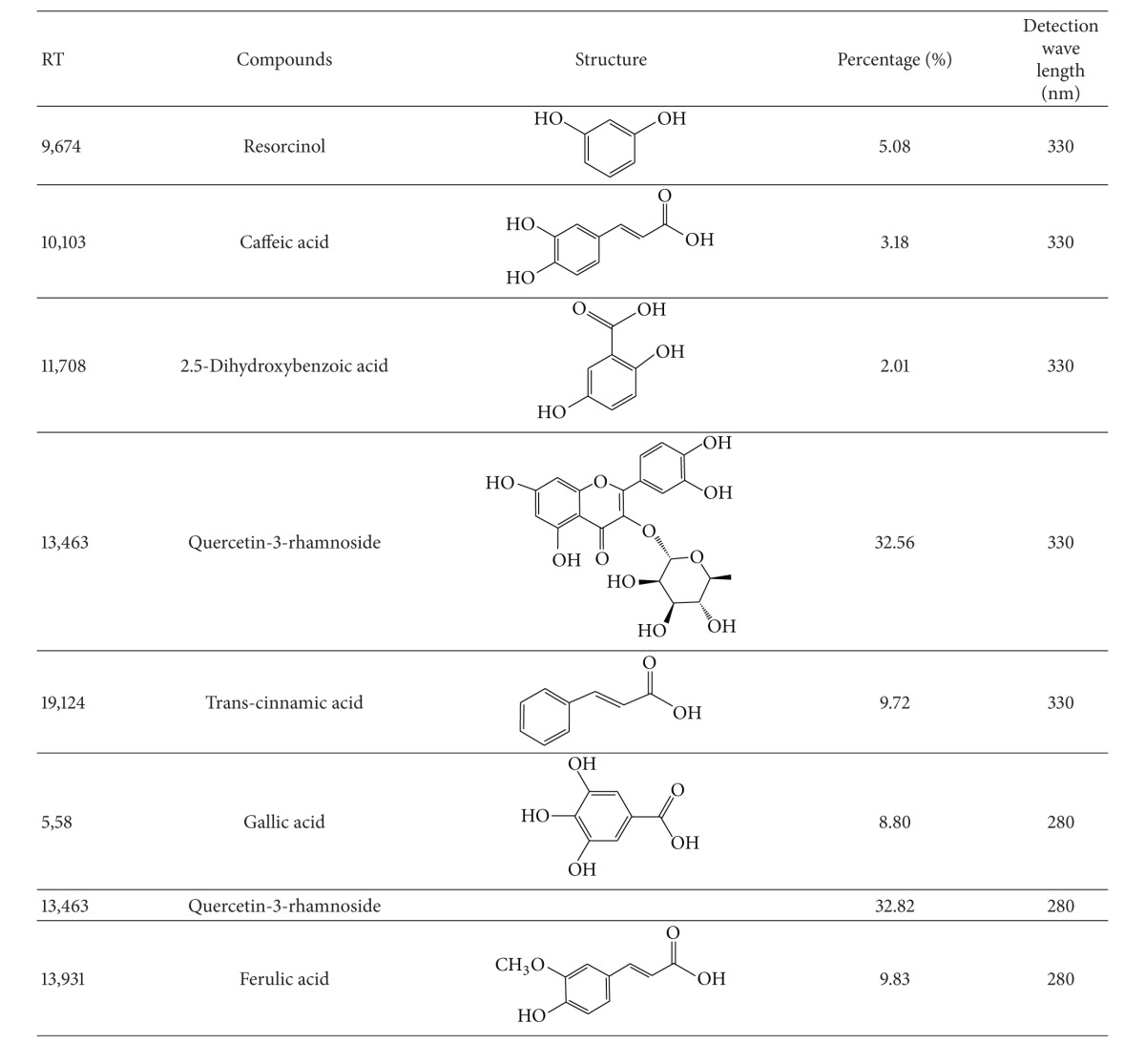

**Table 2:** *Effect of CSEE on DOP, ADR, and NAD levels (ng/mL) in the brain of ICR mice*. Mice fed with 100 and 200 mg/kg of CSEE for 14 days were sacrificed by dislocation of the cervical spine one day after the last behavioral test, and then the brain samples were rapidly washed with PBS (−) and stored at −80°C until use. Monamine neurotransmitters levels were quantified using ELISA kit as explained in Section 2.

Groups	TST			FST		
	DOP	ADR	NAD	DOP	ADR	NAD
Control	5.138 ± 0.07	0.365 ± 0.06	25.196 ± 0.05	6.147 ± 0.02	0.238 ± 0.07	45.655 ± 0.03
Imipramine	6.395 ± 0.08**	0.59 ± 0.03*	51.247 ± 0.04**	6.713 ± 0.06**	0.586 ± 0.02**	50.822 ± 0.05
100 mg/kg	6.378 ± 0.08**	0.551 ± 0.03*	48.640 ± 0.07**	6.717 ± 0.02**	0.584 ± 0.08**	52.167 ± 0.04
200 mg/kg	6.427 ± 0.02**	0.504 ± 0.02*	53.696 ± 0.03**	6.655 ± 0.06**	0.599 ± 0.02**	49.020 ± 0.06

With, DOP: dopamine, ADR: adrenaline, and NAD: noradrenaline.

Each value represents the mean ± SD (*n* = 4). *P < 0.05, **P < 0.01 versus Control group (Student's *t*-test).
